# Short term clinical outcomes of the combined eyeWatch™ implant and eyePlate in refractory glaucoma—a United Kingdom dual centre study

**DOI:** 10.1007/s10792-025-03573-8

**Published:** 2025-06-24

**Authors:** Andrew J. Swampillai, Leon Au, Kin Sheng Lim

**Affiliations:** 1 KCL Frost Eye Research Department, St Thomas’ Hospital, Westminster Bridge Road, London, SE1 7EH UK; 2 Manchester Royal Eye Hospital, Manchester University NHS Foundation Trust, Manchester, UK

**Keywords:** Glaucoma, Intraocular pressure, Hypotony, Aqueous tube shunt, Glaucoma drainage device

## Abstract

**Purpose:**

The eyeWatch™ implant is a novel glaucoma drainage device allowing for a titratable aqueous humour outflow via an external magnetic control unit, facilitating a more controlled reduction in intraocular pressure. We report on 12 month outcomes evaluating the safety and efficacy of eyeWatch™ implant combined with an eyePlate (eyeWatch™ system) in refractory glaucoma.

**Methods:**

Prospective study including patients aged 18 years or older with refractory primary open angle, pigmentary or pseudoexfoliative glaucomas with elevated intraocular pressures > 20 mmHg and previous failed filtration surgery were included. Patients underwent implantation of the eyeWatch™ system. Outcomes were analysed post operatively on 1 day, 1 week, 2 week, 1 month, 2 months, 3 months, 6 months and 12 months after surgery. Main outcome measures were intraocular pressure reduction by ≥ 20% or intraocular pressure < 21 mmHg and no intraocular pressure < 6 mmHg on two consecutive visits after 12 months. Secondary outcomes were mean visual acuity, intraocular pressure and anti-glaucoma medications usage.

**Results:**

Twenty-seven patients were enrolled with a mean age of 68.9 ± 9.5 years. At 12 months, mean reduction of IOP was from 24.4 ± 5.2 mmHg at baseline to 13.2 ± 4.8 mmHg (*p* < 0.01). The number of anti-glaucoma medications significantly decreased from 3.1 ± 0.7 (baseline) to 1.1 ± 1.1 (*p* < 0.01). Visual acuity remained stable for almost all patients from 0.37 ± 0.32 to 0.39 ± 0.26 (logMAR). Complete success rate (intraocular pressure within defined range without glaucoma medications) was 47% and overall success rate was 93%.

**Conclusions:**

The eyeWatch™ system allows for peri and postoperative non-invasive adjustments to resistance of aqueous humour outflow. At 12 months the majority of patients experienced adequate intraocular pressure control, with over 47% not requiring anti-glaucoma medications.

## Introduction

Glaucoma drainage devices (GDD) are now utilised in the management of refractory glaucoma, where traditional filtration surgery has failed [1]. The two most commonly used devices in current practice are the Ahmed glaucoma valve (New World Medical Inc., CA) and Baerveldt glaucoma implant (Johnson & Johnson Surgical Vision Inc., CA). The Ahmed glaucoma valve (AGV) implant consists of a 184 mm^2^ plate with an internal flow restrictive system designed to minimize postoperative hypotony and associated complications such as anterior chamber shallowing, choroidal detachment or suprachoroidal haemorrhage [2–4]. The Baerveldt glaucoma implant (BGI) is a non-valved device, available in two different end plate sizes (250 and 350 mm) [5, 6]. It requires restriction of flow through use of either an intraluminal suture or tube ligation enabling formation of an adequate capsule [3].

While comparative studies have demonstrated the BGI to lower intraocular pressure (IOP) greater in comparison to the AGV [3, 7], several limitations to these interventions still persist. One of the main drawbacks is the unpredictability in IOP control during the early postoperative period. Flow restriction required in BGI often results in a hypertensive phase, necessitating the need for additional anti-glaucoma medications. A significant risk of hypotony when the ligation suture dissolves or after the removal of the intraluminal suture [8], along with significant variability in ‘opening’ pressures in AGV necessitating testing prior to implantation [9].

**Fig. 1:**
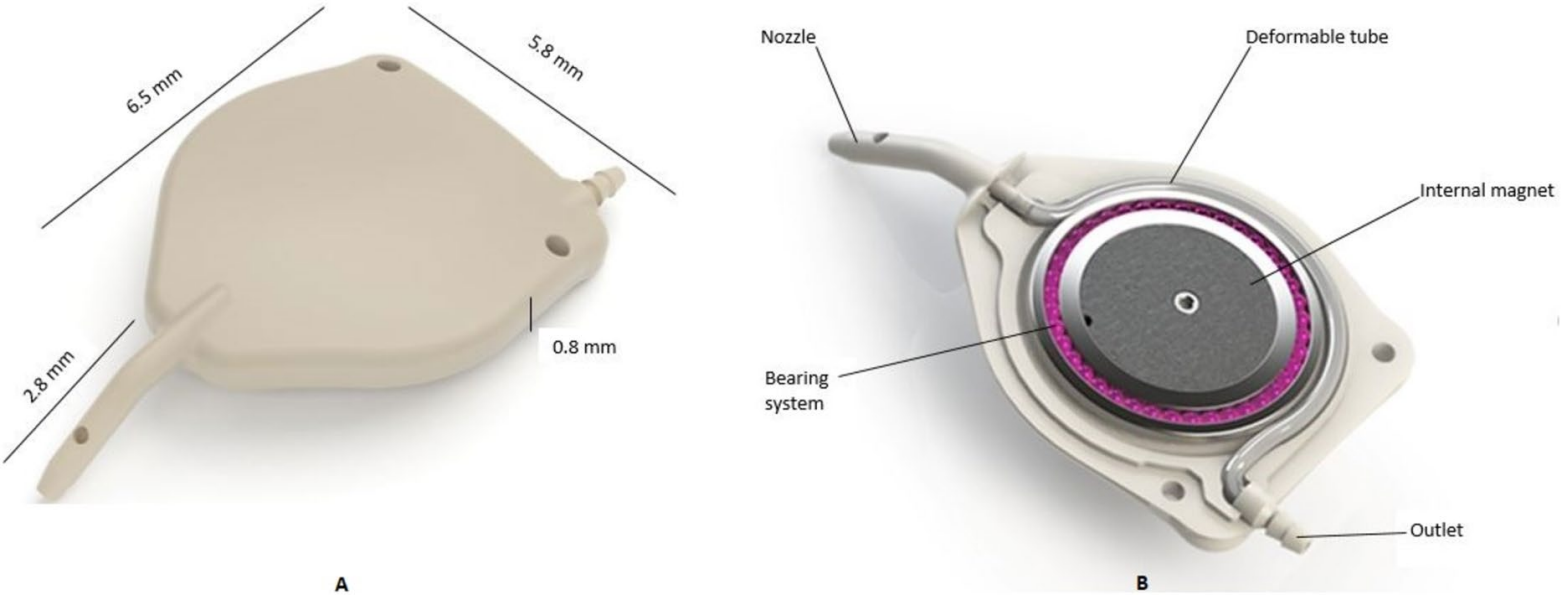
Dimensions of the eyeWatch™ device illustrating the internal compressible tubing mechanism

The eyeWatch™ system (eW) (Rheon Medical, Switzerland) is a novel GDD customised to provide predictable and more precise IOP control in the early postoperative period. This builds upon the original eyeWatch™ device, which allows for a titratable resistance to aqueous outflow from the anterior chamber. This device has been validated in vitro [10] and in vivo [11]. The design has been described elsewhere [12] but a brief description follows. The eW device comprises of an implant containing a deformable silicone tube to drain aqueous from the anterior chamber. The implant is made out of Polyetheretherketone (Victrex, Lancashire, UK). The deformable tubing is made out of medical grade silicone (MED-4830, Nusil Technology, PA, USA) and has a lumen of 0.2 mm for a length of 17 mm. The silicone tubing is situated around an internal magnetic disc, which is surrounded by ball bearings facilitating its rotation and a ring on top of the tubing. This ring allows compression and decompression of the tubing, corresponding to a controlled increase or decrease in resistance to aqueous outflow. (Fig. 1).

**Fig. 2:**
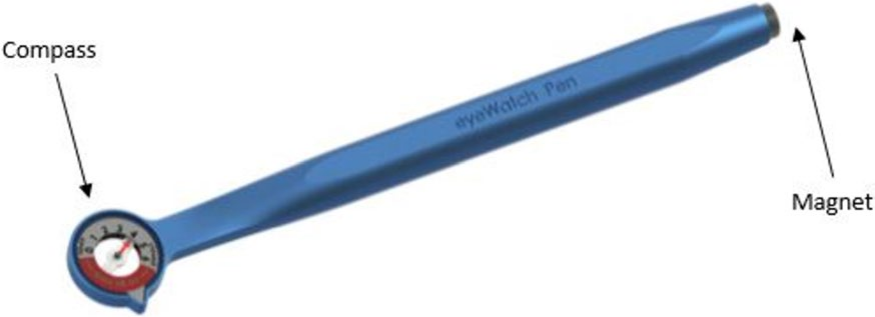
The eyeWatch™ pen displaying the compass assessing internal rotator position at one end and the magnet to rotate internal motor at the other

During the intraoperative and postoperative period, the modification of IOP can be performed in a non-invasive manner by altering the rotor position by use of a purpose designed eyeWatch™ Pen (Fig. 2).

**Fig. 3:**
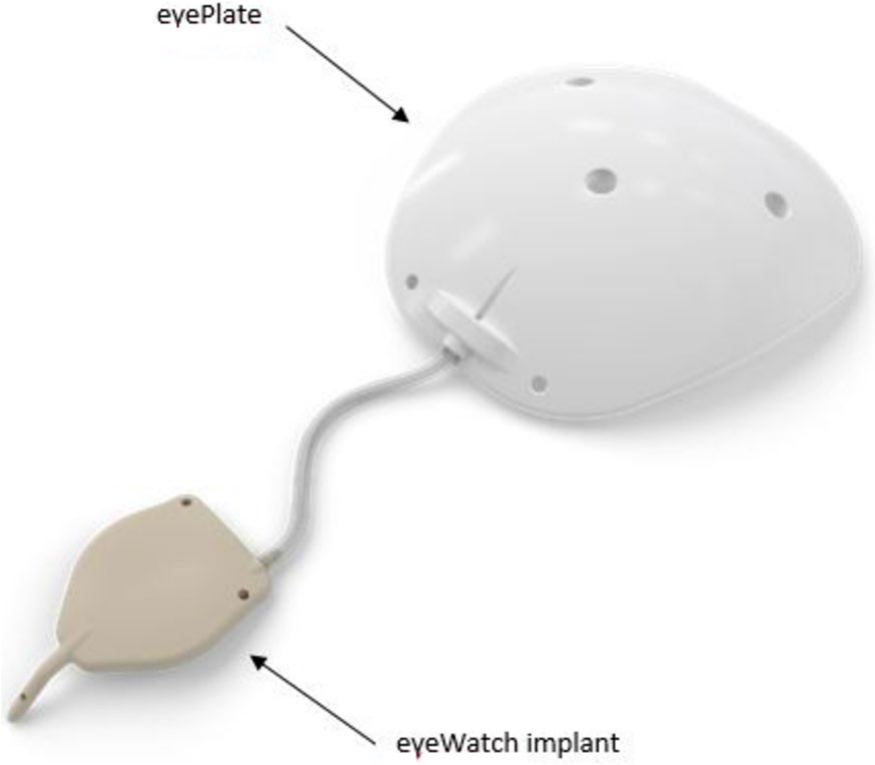
The eyePlate conncted to the eyeWatch implant (eyeWatch™ system)

Previous studies of implant have studied this device connected to a standard 250 or 350 BGI plate [11, 13]. The company have since developed their own plate design called the “eyePlate” (Fig. 3). The eyePlate is a non-valved plate made of medical grade silicone (Avantor, Nusil Technology, PA, USA). The plate exists in two sizes (eyePlate-200 and eyePlate-300) with areas of 200 mm^2^ and 300 mm^2^ respectively. The eyePlate-300 was used in this study and its dimensions are 18.9 mm long, 18.5 mm wide with a thickness of 0.8 mm. The plate features two fixation holes for scleral attachment and three fenestrations for limiting the bleb’s volume after implantation. The seton tube is similar in both versions of the eyePlate and has a length of 28 mm, an external diameter of 0.63 mm and an internal lumen of 0.30 mm.

This prospective study aimed to evaluate the clinical outcomes of this new system in patients with uncontrolled glaucoma and prior failed filtration surgery.

## Methods

Ethics committee approval was obtained from the London—Dulwich research ethics committee (REC no: 18/LO/0190). This study protocol adhered to the Declaration of Helsinki. Eligible patients were recruited in clinic and each patient voluntarily gave written informed consent to participate in the study. The study is registered at clinicaltrial.gov (NCT03210571).

### Inclusion and exclusion criteria

Patients aged 18 years or older with refractory primary open angle (POAG), pseudoexfoliative (PXF) or pigmentary (PG) glaucomas after previous failed filtration surgeries and who were candidates for a GDD were consecutively included. Patients with the diagnosis of angle closure glaucoma, secondary glaucomas (e.g. neovascular, uveitic, silicone oil, congenital), angle dysgenesis, microphthalmia, optic neuropathy secondary to other causes than glaucoma, diabetic retinopathy graded more than severe non-proliferative, previous strabismus or corneal transplantation surgery, previous retinal detachment or pars plana vitrectomy surgery and history of ocular trauma were all excluded. Patients unable to complete the required post-operative visits were also excluded.

### Surgical technique

**Fig. 4:**
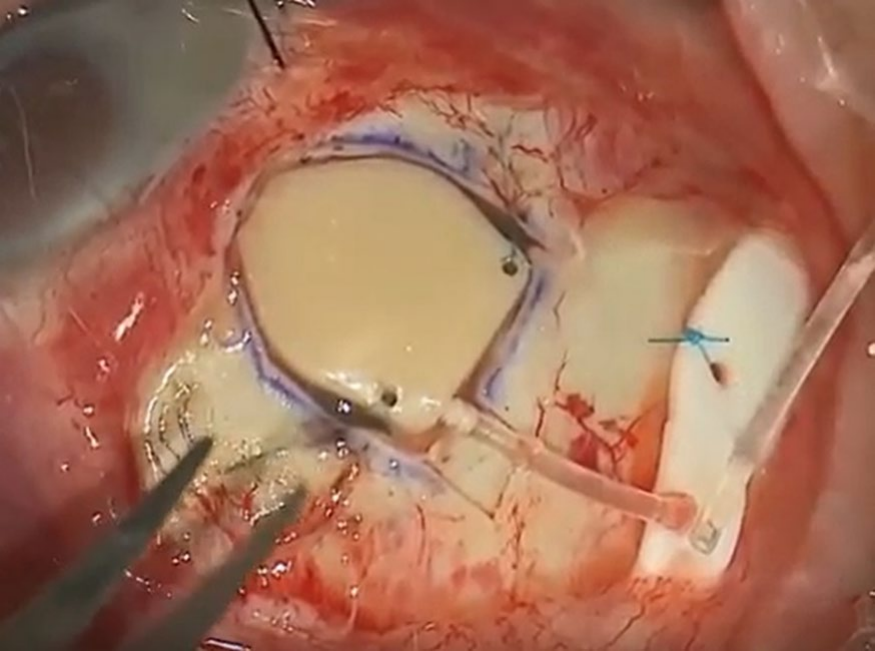
Implantation of the eyeWatch™ system

All surgeries were performed by site investigators (LA or KSL) under local or general anaesthesia. Superior temporal placement was the preferred position of the eW (Fig. 4).

A fornix-based peritomy was made at the superior temporal limbus, along with exposure of the sclera by careful dissection of the Tenon’s capsule. Gentle wet-field cautery was used to achieve haemostasis. The eyePlate was placed 10–12 mm from the limbus, between the rectus muscles and secured with two single Prolene 7–0 sutures. A 25-gauge needle was used to fashion a track, enabling the device’s nozzle to be inserted into the anterior chamber. A superficial sclerectomy was performed using the outline of the eW device 2 mm from the limbus, enabling the device to be fixed securely using two single Nylon 9–0 sutures. The eW was initially delivered on the opened position (position 0). The magnet was then closed (position 6) in order to increase the fluidic resistance. The eyePlate tube was trimmed appropriately and fixed to the eW device. A pericardium patch (Tutoplast, Tutogen Medical GmbH) of about 6 × 6 mm was finally secured on the eW device using interrupted Nylon 10–0 sutures, with conjunctival closure using interrupted Vicryl 8–0 sutures. Patients administered a post-operative treatment regime consisting of topical antibiotics (Chloramphenicol 0.5% preservative free, Bausch & Lomb, UK) four times a day for two weeks and corticosteroids (Dexamethasone 0.1% preservative free, Rayner Pharmaceuticals, UK) every two hours for the first month; tapered in a reducing dose over the next two months.

### Follow-up and study measurements

Follow-up visits were performed at day 1, week 1, week 2, month 1, month 2, month 3, month 6 and month 12 after surgery. Best-corrected distance visual acuity (BCVA) was assessed using Logarithm of the Minimum Angle of Resolution (logMAR). Pachymetry was performed using a non-contact pachymeter (Pachmate 2; DGH Technology, Pennsylvania, USA) and corneal endothelial cell density using specular microscopy (EM 3000, Tomey, Arizona, USA). All patients underwent slit lamp biomicroscopy, Goldmann applanation tonometry, gonioscopy and optic disc evaluation where appropriate.

**Fig. 5:**
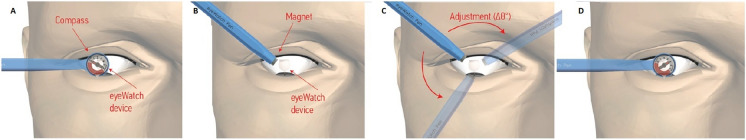
Adjustment of the eyeWatch™ system: **a**—positional outflow measured using the compass of the eyeWatch Pen. **b**—eyeWatch™ Pen turned around to reveal external magnet. **c**—EyeWatch™ Pen turned in direction desired. **d**—New position of the eyeWatch™ implant confirmed with the eyeWatch™ Pen compass

Goldmann tonometry and reading of the eW position was performed by the site investigator who performed the procedure (LA or KSL). The eW implant was adjusted in one of two ways: if IOP was raised above 16 mmHg, the rotor was adjusted to decrease outflow resistance. Alternatively, if the IOP was low (i.e. < 6 mmHg), the rotor was adjusted to increase outflow resistance and increase IOP to the desired value. The method of adjustment of the rotor with the eyeWatch™ Pen (Fig. 5) has previously been described [12].

### Outcome measures

The outcome measures used in this study were similar to those in previous studies evaluating the implant connected with BGI plate [11, 13]^11 13^ and are summarised below:

1. The primary outcome measures obtained were IOP, BCVA, glaucoma topical medications and number of complications.
2. The primary endpoint was the ability of the eyePlate combined to an eW implant to reduce IOP by ≥ 20% or IOP < 21 mmHg, with no IOP < 6 mmHg on two consecutive visits after 12 months. A complete success was classified as an IOP ≤ 18 and ≥ 6 mmHg with a reduction by 20% from baseline at the last follow-up visit, without the need for glaucoma medications. Overall success was classified as an IOP ≤ 21 and ≥ 6 mmHg and a reduction by 20% from baseline at the last follow-up visit with the use of glaucoma medications.
3. Failure was defined as need for additional glaucoma surgical intervention, loss of light perception, explanting of device, IOP > 21 mmHg or < 20% reduction from baseline on 2 visits after 3 months or persistent hypotony (IOP < 6 mmHg on 2 consecutive study visits after 3 months). Additional glaucoma surgical intervention was defined as the requirement for a further filtering procedure or revision to be performed in theatre. Slit lamp interventions e.g. paracentesis or reformation of anterior chamber were not considered to be in this category.

### Statistical analysis

Data were processed as mean ± SD or as percentages where appropriate. Normality of data was assessed by Shapiro–Wilk test. Comparison of pre and post-operative data was processed via paired *t*-test for parametric and Wilcoxon signed-rank test for non-parametric distributions. All statistical analyses were performed using Statistical Package for Social Sciences (SPSS) version 26 (IBM Corp, Chicago, USA). A *p*-value of ≤ 0.05 was considered statistically significant.

## Results

Table 1 summarises the baseline characteristics of patients. Initially 30 patients were screened and fulfilled the criteria for inclusion. Two patients withdrew consent after personal consideration and one patient died after recruitment and before surgery was planned. A total of 27 patients were recruited between June 2018 and October 2020. Mean age was 68.9 ± 9.5 years. Type of glaucoma enrolled were 22 POAG (81%), 4 PXF (15%) and 1 PG (4%). Caucasians (55%) were the most prominent group treated, typically representing the population present in Western Europe. Sixty three percent (63%) of the patients were men. Statistical difference between gender groups was not significant (p < 0.05).

**Table 1:** Baseline demographics and characteristics

Baseline	eyeWatch system (n = 27)
Age (years), mean ± SD	68.9 ± 9.5
*Gender, n (%)*	
Male	17 (63)
Female	10 (37)
*Ethnicity, n (%)*	
Caucasian	15 (55)
Asian	2 (8)
African	6 (22)
Others/unknown	4 (15)
*Type of glaucoma, n (%)*	
POAG	22 (81)
PEX	4 (15)
PG	1 (4)
IOP (mmHg), mean ± SD [range]	24.4 ± 5.2 [18 to 38]
Glaucoma medications, mean ± SD [range]	3.1 ± 0.7 [1 to 4]
Snellen BCVA Mean ± SD [range]	0.4 ± 0.3 [0 to 0.7]

SD, standard deviation; N, number of patients

**Fig. 6:**
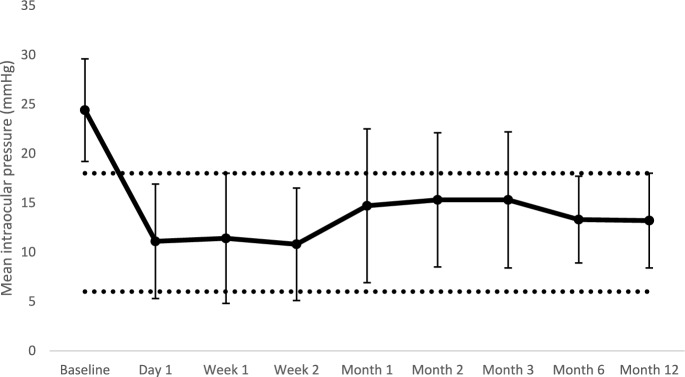
Mean IOP at each study visit (black circle) with standard deviations (bars). The area between the dotted lines represents the desired IOP range (18 ≤ IOP ≤ 6 mmHg) as defined by the primary endpoint

The mean IOP at baseline was 24.4 ± 5.2 mmHg and was decreased to 13.2 ± 4.8 mmHg at 12 months (*p* < 0.001, paired t-test) amounting to a 45% reduction from baseline (Fig. 6). All patients experienced a reduction of at least 20% of the IOP value or did have an IOP < 21 mmHg at their last visit follow-up. Only two patients experienced an IOP ≥ 21 mmHg on two consecutive visits after 1 month of follow-up (7%). None of the patients have experienced hypotony (IOP < 6 mmHg) after 30 days post-operatively. At 12 months, the complete success rate (IOP within the defined range and no glaucoma medication needed) was 47% and overall success rate was 93%.

The use of topical glaucoma medications significantly decreased from 3.1 ± 0.7 (baseline) to 1.1 ± 1.1 at 12 months (*p* < 0.01, paired t-test). Visual acuity remained stable for almost all patients with baseline acuity at 0.37 ± 0.32 and 0.39 ± 0.26 at 12 months (logMAR). The mean corneal endothelial cell density of the 27 patients recruited was 1997 ± 584 cells/mm^2^ at baseline pre-operatively (CoV 0.29) and 1923 ± 609 cells/mm^2^ at 12 months (CoV 0.31); equating to a loss of 5.5%. Mean pachymetry demonstrated stability with 530 ± 66 μm pre-operatively and 551 ± 93 μm at 12 months (increase of 4.3%). Adjustment of the eW was mostly done within the first 2 months post-op (Table 2). After this period, the implant is left in its fully open position (position ‘0’).

**Table 2:** Indicated positions and number of adjustments with the eyeWatch™ Pen

Visit	Day 1	Week 1	Month 1	Month 2	Month 3	Month 6	Year 1
Mean position † indicated on eyeWatch™ Pen	5.3	4.8	3.5	1.8	1.3	1.2	1.2
Number of eyeWatch™ implants adjusted	7	10	9	9	6	1	0

^†^Positions indicated on the eyeWatch™ Pen go from 0 indicating no resistance to outflow to position 6 indicating maximal resistance

All complications and interventions were recorded for each post-operative visit. There was a total of 16 complications affecting 14 patients including mild choroidal detachment, shallow anterior chambers and hyphaema (Table 3). Most of these complications were resolved spontaneously, while others required minor surgical revision (Table 4). Device failures (defined as a failure of the device without any related adverse event or consequence to the patient) occurred in one patient, where the implant was found to have a blocked mechanism (3%). The device was not able to be adjusted anymore with the eyeWatch™ Pen. Follow-up of this case was performed according to the protocol, with no serious adverse event occurring for the patient.

**Table 3:** Number of complications after the eyeWatch™ device implantation

eyeWatch complications	eyeWatch system (n = 27)
*Early (*< *3 month)*	
Transient choroidal detachment	3 (11%)
Flat/shallow chamber	5 (18%)
Wound leak	1 (3%)
Hyphaema	2 (7%)
Retinal detachment	1 (3%)
Iris incarceration	1 (3%)
*Late (after 3 months)*	
Transient diplopia	1 (3%)
Cataract formation	1 (3%)
Uveitis	1 (3%)

**Table 4:** Number of overall surgical interventions after the eyeWatch™ system implantation

eyeWatch surgical intervention	eyeWatch system (n = 27)
Corneal graft	1 (3%)
Vitrectomy	1(3%)
Anterior chamber reformation	1 (3%)
Laser iridectomy	1 (3%)
Tube obstruction—ab interno fibrin removal	2 (7%)
Device explanted	1 (3%)

## Discussion

This study reports on the first clinical outcomes of the eW implant combined with the specifically designed eyePlate 300. The goal of the eW system is to enable customised adjustments of aqueous humour outflow, thus minimising the risks of hypotony or inadequate IOP reduction. During the early post-operative period, the eW system efficiently controlled the IOP to avoid the risk of hypotony as well as avoiding pressure spikes. The adjustments of the eW were performed in a non-invasive manner; with the advantages of being time efficient for the clinician and atraumatic and with comfortable for the patient.

Treatment success was subdivided into complete and qualified success on the basis of final IOP at 12 months, number of topical glaucoma medications used, loss of light perception and need for additional glaucoma surgical interventions. In this study 47% of patients experienced complete success and 93% partial success at one year, thus demonstrating the eW to both safe and efficient in managing refractory glaucoma. This success rate is higher than reported for the BGI [3, 8, 13]. A recent study evaluating the eW system with both eyeplate 200 and 300 showed a similar rate of complete success at 12 months [14]. The final number of topical glaucoma medications required at 12 months (1.1 ± 1.1) was also significantly better than the BGI group (2.1 ± 1.4) at a similar time point in the Primary Tube Versus Trabeculectomy study [15].

The ability to allow for dynamic modification of outflow is potentially favourable to the need for additional aqueous suppressant medications in the hypertensive phase encountered in other GDD [16] and/or the need for an intraluminal suture in non-valved GDD with subsequent removal as a secondary procedure [17, 18]. Aqueous outflow resistance of the eW device is initially set to close to position 6 to avoid hypotony, indicating a highly resistive tube. Conversely in later stages, when a fibrotic capsule has formed around the eyePlate providing enough resistance, the implant was set to open position (readings 0–1) allowing passage of aqueous through the device. By using the eW, the potential for postoperative IOP spikes are avoided with the non-invasive adjustments made on the device, thus reducing the need for topical aqueous suppressants or anti-fibrotic agents.

Compared to a BGI 350, the eyePlate 300 is less wide (18.5 mm vs 32 mm) but longer (18.9 mm vs 14 mm). This geometrical difference may confer some advantages compared to the design of the Baerveldt plate; the eyePlate is designed to be situated in between the extraocular rectus muscles, while the BGI requires placement under these. Consequently there is no need to grasp or stretch the rectus muscles to insert the eyePlate, which also minimises the risk of having ocular-cardiac reflexes. This reflex can result in bradycardia or can increase the risk of arrhythmia in patients with heart conditions. Some surgeons prefer performing implantation of a BGI under general anaesthesia for this reason [19, 20]. It might be theorized magnetic resonance imaging is a concern for patients implanted with eW. During our study, two patients underwent such imaging for reasons unrelated to the study. Both patients were found to have not experienced any problems during and after this imaging procedure. No artefacts interfering with the imaging diagnostics were reported. The clinical efficacy and integrity of the devices in these patients were not affected or altered either. A previous report has confirmed these observations [21].

In our study, the failure rate was 7% with 2 patients experiencing IOP above the defined limit (21 mmHg) for 2 consecutive postoperative visits after 1 month. None of these patients required secondary surgical intervention and this compares favourably with previous studies with failure rates reported to be ranging from 13 to 28% [3, 8, 15]. Most complications reported in the first 3 months resolved spontaneously. Thirteen out of 16 complications reported did happen during the early post-operative period. Most of them (77%) are mostly related to the surgical procedure rather than to the device itself. One case of shallow anterior chamber was found in a patient, despite a good IOP (8 mmHg). One device failure was found due to a blockage of the rotating mechanism not amenable to adjustment with the eyeWatch™ pen; this was later explanted and the patient treated medically. One patient required vitrectomy for retinal detachment, which was not thought to be attributed to the device itself in the opinion of the chief investigators. Another underwent an endothelial keratoplasty for corneal decompensation: this patient was noted to have a low endothelial cell density (~ 1000 cell/μm^2^) before involvement in the study. Mean endothelial cell density showed a 5.5% loss at 12 months, which is much lower than studies evaluating endothelial cell loss in BGI (13.1% [22]) and AGV (10.5% [23] and 12.3% [24]).

We do acknowledge some limitations to this study. The relatively small cohort of patients (n = 27) and study duration (12 months) could limit the significance of the results. Further studies with an increased number of patients and extended follow-up time are required. Although the results presented are not derived from a single surgeon, it may be argued these are more applicable to real world outcomes. A limitation of the device itself is the requirement for the patient to wait after adjustments made by the eyeWatch™ pen, which theoretically adds more time to post-operative visits.

In summary, this study reports on the clinical outcomes of the eW. This novel GDD allows for performing peri and post-operative non-invasive adjustments to titrate aqueous outflow. Such precise adjustments help in reducing the risk of hypotony and related complications, frequently encountered in other GDD during the early post-operative phase. The eW also maintained target IOP levels with a significantly reduced glaucoma medical therapy and without the need of additional filtering surgical re-interventions. After 12 months of follow-up, 93% of the patients experienced a successful outcome, with 47% not requiring any additional glaucoma medications for maintenance of targeted IOP.

## Data Availability

No datasets were generated or analysed during the current study.
